# Intermediated Reality: A Framework for Communication Through Tele-Puppetry

**DOI:** 10.3389/frobt.2019.00060

**Published:** 2019-07-23

**Authors:** Llogari Casas, Kenny Mitchell

**Affiliations:** School of Computing, Edinburgh Napier University, Edinburgh, United Kingdom

**Keywords:** augmented reality, collaborative mixed reality, real-time graphics, human-computer interaction, tele-puppetry

## Abstract

We introduce *Intermediated Reality* (IR), a framework for intermediated communication enabling collaboration through remote possession of entities (e.g., toys) that come to life in mobile Mediated Reality (MR). As part of a two-way conversation, each person communicates through a toy figurine that is remotely located in front of the other participant. Each person's face is tracked through the front camera of their mobile devices and the tracking pose information is transmitted to the remote participant's device along with the synchronized captured voice audio, allowing a turn-based interactive avatar chat session, which we have called *ToyMeet*. By altering the camera video feed with a reconstructed appearance of the object in a deformed pose, we perform the illusion of movement in real-world objects to realize collaborative tele-present augmented reality (AR). In this turn based interaction, each participant first sees their own captured puppetry message locally with their device's front facing camera. Next, they receive a view of their counterpart's captured response locally (in AR) with seamless visual deformation of their local 3D toy seen through their device's rear facing camera. We detail optimization of the animation transmission and switching between devices with minimized latency for coherent smooth chat interaction. An evaluation of rendering performance and system latency is included. As an additional demonstration of our framework, we generate facial animation frames for 3D printed stop motion in collaborative mixed reality. This allows a reduction in printing costs since the in-between frames of key poses can be generated digitally with shared remote review.

## 1. Introduction

Mediated Reality (MR) after (Stratton, 1896) captures the concept of transforming one's sensory experience of the real world imperceptibly through artificial means and was first practically demonstrated in a modern context by Mann (2002). Mixed Reality (MR) environments, defined by Milgram et al. (1994) are those in which real and virtual world objects are presented together in the same place and time. Frequently, such environments tend to be accessible only by a single person, the one interacting with the device. This method of interaction, however, differs greatly from our society. We, as individuals, generally interact and collaborate with other human beings on daily basis. This urged (Billinghurst and Kato, 1999) to define what is known as Collaborative Mixed Reality (CMR), which we build upon by means of tele-present augmented reality to introduce an Intermediated Reality (IR) framework. This approach aims not only to allow users to collaborate remotely in a novel way, but also to enhance creativity, imagination and interaction with inanimate objects of our daily lives. In this sense, we propose an Augmented Reality system capable of animating real world objects and toy figures with photo-realistic results. The research presented in this manuscript allows participants to interact with inanimate objects and toys as if they were brought to life.

CMR systems allow multiple users to access the same shared Mixed Reality environment. This allows a more natural approach to mediated social interaction, in which multiple users can collaborate and interact with each other through digital displays in a shared space. For instance, such a concept has been demonstrated in the *AR Travelers* game within the Augmented Creativity framework (Zünd et al., 2015), or for wider scale distributed collaborative settings (Demir and Karaarslan, 2018). In this work, we introduce a mediated communication framework for organized development of IR systems across the Reality-Virtuality continuum (Milgram et al., 1994).

Our ToyMeet practical demonstration focuses on mixing real and virtual spaces seamlessly in a remote shared context. We extend existing frameworks for communication (Shannon and Weaver, 1949) and interactive systems from Kennedy et al. (1996) and Kransner and Pope (1988) noting the context of many practical variations of architectural patterns (Buschmann et al., 2007) to adapt these models in our Intermediated Reality communication scenario. We follow (Konrad et al., 2017) to define a low-latency framework for broadcasting visual and audible cues efficiently.

**Figure 1:**
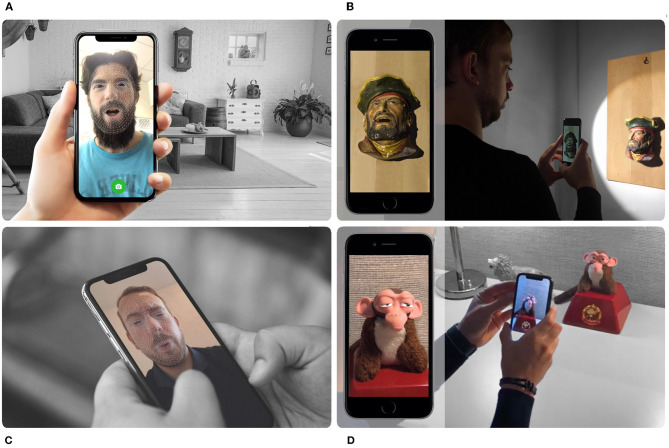
Multiple participants using Intermediated Reality (IR) as a method of collaboration and entertainment through the remote possession of toys that come to life in Augmented Reality (AR). **(A)** Participant A recording a message containing voice audio with synchronous facial expressions. **(B)** Participant B reproducing the sender's message through a real-world toy in augmented reality. **(C)** Participant B recording a reply message containing voice audio with synchronous facial expressions. **(D)** Participant A reproducing the reply from his previous message through a real-world toy in AR.

By augmenting the camera feed with our reconstructed appearance of the object in a deformed shape, we perform the illusion of movement for real-world static objects, remotely. As part of a two-way conversation, each person communicates through a toy figurine that is remotely located in front of the other participant (see Figure 1). Each person's face is tracked through the front camera of their mobile devices and the tracking pose information is transmitted to the remote participant's device along with the synchronized voice audio, allowing a turn-based interactive avatar chat.

*Intermediated Reality* also facilitates the practical application of collaborative workflows, for example in Stop Motion Animation. The IR system allows remote collaboration and rapid posing of digital character meshes through facial blend shapes acquired instantaneously by mobile devices. This can be subsequently used to generate in-between 3D printed key frames and so animates puppet expressions for Stop Motion Animation directly from a participating hand-held device. We highlight the main contributions of our research as follows:

- we introduce, *Intermediated Reality* (IR), a tele-present augmented reality framework that enables mediated communication and collaboration for multiple users through the remote possession of toys brought to life.
- inspired by the *Shannon and Weaver (**1949**) model*, we define the tele-puppetry model of communication for delegated interaction in IR.
- we describe how the voice and facial expressions of the sender are captured and synchronized, and how these, are broadcast and reproduced remotely on an enlivened toy with single and multiple participants.
- we demonstrate the application of the IR framework to Stop Motion Animation production work-flows by directing the facial expressions of a character remotely from a mobile phone.
- we validate the media richness effectiveness of *Intermediated Reality* through a comparative user study and a system usability scale.

## 2. Related Work

*IR* relates to a variety of research areas in Augmented Reality to integrate a Collaborative Mixed-Reality (CMR) system (Billinghurst and Kato, 1999). In practice, we draw from *Image Retargeting* techniques to animate physical puppets (Casas et al., 2017), and shadows (Casas et al., 2018) (section 2.1). The collaborative system bases its structure on related frameworks in mixed reality and graphical user interfaces as detailed in section 2.2. IR relates to physical avatars and robots to support remote collaboration in a mediated communication environment as explained in section 2.3.

### 2.1. Retargeting Reality

The seamless integration between computer-generated objects with real-world scenes is desirable in Augmented Reality applications. To achieve this, virtual objects need to blend perfectly with the real-world environment. Madsen et al. (2003) defined the consistency of the geometry, the coherence of lighting and the consistency of time as key aspects necessary for photo-realistic rendering in Augmented Reality (AR).

Retargeting approaches for AR are relatively novel and the first method was only introduced in 2011 by Leão et al. (2011). Prior to this, live virtual objects have been typically overlaid into the real-world as an independent entity. Such methods provided a pipeline in which a deformed mesh was projected on top of a real-world cube to create an illusion of deformation. Following this line of work, Takeuchi and Perlin (2012) introduced *Clayvision*, a related scheme for animating real-world objects in the city. This method was constrained to a very limited set of locations and conditions. To solve for a broad set of objects, Casas et al. (2017) introduced a flexible method for object appearance retargeting using texture deformation from a live video stream. This approach allows for plausible photo-realistic Augmented Reality appearance, since the material, illumination and shading of the object are updated to the virtual mesh in each frame. This method is used in our *ToyMeet* demonstration (section refsystemOverview) for bringing to life inanimate real-world toys.

Additionally, to achieve consistency of lighting, the proper shading properties need to be found so that a computer-generated object looks just like a tangible object in the real-world scene. Hence, to achieve such photo-realistic appearance, a virtual object must project a correct shadow in a given environment. Following a retargeting approach, Casas et al. (2018) introduced *Shadow Retargeting*. This method synthesizes virtual shadows directly by sampling the image that contains the shadow of the toy presented in the real-world. As the virtual mesh is animated, this approach warps the real shadow to retarget its appearance to the virtual one. Where the warping samples an area outside the shadow, the appearance gets reconstructed using a search for sampling candidates. *Shadow Retargeting* is used in *ToyMeet* for bringing to life with photo-realistic appearance inanimate physical toys.

### 2.2. Collaborative Mixed-Reality Systems

Billinghurst and Kato (1999) defined Collaborative Mixed Reality (CMR) systems as a natural medium for computer supported collaborative work (CSCW). Rekimoto (1996) and Billinghurst et al. (1998) proposed early examples of MR cooperation with face-to-face experiences using hand-held and Head Mounted Displays (HMD). Kiyokawa et al. (2002) further concluded that AR could significantly improve collaboration within users by merging the real and virtual world into the same shared context. This shared MR context allowed to use the same non-verbal cues used in face-to-face conversations, while also interacting with the AR content overlaid in front of them. More recently, Zünd et al. (2015) proposed a system in which multiple collaborators could simultaneously enhance creativity in AR. Fairchild et al. (2017) and Steed et al. (2012) embody processes of capturing remote participants beamed into the shared space with visual depictions of them displayed digitally in mixed reality. Our system provides a real-world physical intermediary for the remote person's presence with animated expression synchronized with transmitted vocal audio.

While research on CMR systems has focused primarily on real-time collaboration, less work has been done on the use of AR for asynchronous participation. Renevier and Nigay (2001) introduced an early CMR system that allowed the creation and visualization of AR messages in real-world space for archaeologists. Such system was recently extended by Nassani et al. (2015) to allow users to place virtual labels on any object or location in the real world. Kooper and MacIntyre (2003)'s work, is another pioneering example of an asynchronous CMR system. This research developed an AR browser that could get registered to a specific real-world location and became visible by other participants.

Related schemes for asynchronous messaging have been introduced by Everitt et al. (2003) and Kjeldskov et al. (2009) using interactive boards. These systems allow users to leave non synchronized messages to other participants. Our research builds on these foundations to create a turn-based interactive avatar chat for tele-puppetry in a tele-present Intermediated Reality system. Each participant first sees his or her own vocalization and facial expressions captured locally, then transmits the message to a database and is finally made available to the receiver's physical puppet when the remote user accesses the chat.

### 2.3. Physical Avatars for Remote Collaboration

The use of physical objects as avatars for remote collaboration has been conceived previously. Sekiguchi et al. (2001) introduced a Robotic User Interface (RUI) for interpersonal communication using robots as physical avatars. In their system, remote users could communicate shapes and movements with each other using snake-like robots equipped with servomotors that responded to information transmitted through a network. This concept was further extended by applying that technique to remote controlled fluffy toys (Sekiguchi et al., 2004). We base our approach on the same bases, triggering the AR animations on real-world according to the information received from the remote participant.

Yim and Shaw (2011) proposed interactive bidirectional robot intermediaries for performing tasks and applications. Our approach draws from this concept to use toys figurines as interlocutors in both ends of a remote communication.

Drawing from Karsch et al. (2011), we aim to create the illusion of movement on static puppets by seamlessly rendering synthetic objects into a real-world scene. Our system brings the advantages of both, distributed and mediated avatars, to propose an Augmented Reality system capable of animating real world objects and toy figures with photo-realistic results. The research presented in this manuscript allows participants to interact with inanimate objects and toys as if they were brought to life.

## 3. Intermediated Reality

*Intermediated Reality* introduces a new method of collaboration and entertainment through the remote possession of toys brought to life in mobile Augmented Reality (AR). Section 3.1 defines the tele-puppetry model of communication for mediated interaction. Section 3.2 analyses the medium effectiveness of Intermediated Reality.

### 3.1. Tele-Puppetry Model of Communication

**Figure 2:**
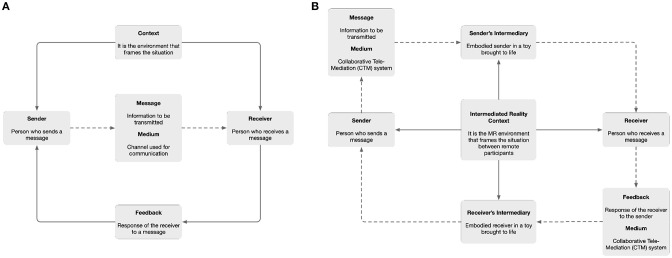
**(A)** Shannon and Weaver model of communication. **(B)** Tele-puppetry model of communication.

Communication is the action of exchanging information between two or more participants in order to transmit or receive messages through a shared system of signs and semantic rules. The *Shannon and Weaver (**1949**) model* (Figure 2A) is specially designed to develop an effective communication between the sender and the receiver. It contains context, sender, message, medium, receiver and feedback as the key components of the model.

- *Context* is the situation in which the communication is developed. It is the set of circumstances that affect both the sender and receiver, and also determine the interpretation of the message.
- *Sender* is the person who transmits a message. This is encoded using a combination of words understandable to the receiver.
- *Message* is the information that is exchanged between the sender and the receiver.
- *Medium* is the channel through which the encoder will communicate the message. This can be printed, electronic or audible and depends on the nature of the message and the contextual factors of the environment.
- *Receiver* is the person who interprets the message. This is influenced by the context to which it is exposed when decoding the message.
- *Feedback* is the response or reaction of the receiver to a message. The communication becomes effective when a response is emitted.

Drawing from the *Shannon and Weaver (**1949**) model*, we define the tele-puppetry model of communication for intermediated interaction in Augmented Reality (Figure 2B). In this approach, we use toys brought to life as a channel to send and receive messages. This means that additional components are now present on the loop of communication. These new elements, which we define as the *sender's intermediary* and the *receiver's intermediary*, are the responsible for embodying and emitting messages using a physical toy brought to life. These components are the real-world representation of the sender or the receiver in a remote venue.

In the tele-puppetry model of communication, the *context* translates into our *Tele-present Mixed Reality* (TMR) context. Both distributed participants share the same MR environment as a tele-present collaborative mixed reality (CMR) system (Billinghurst and Kato, 1999). Further, this context is not only linked with the *sender* and the *receiver*, but also with the *sender's mediator*, as the presenter of the emitted message from the sender in the receiver's location. Additionally, due to the distributed nature of a TMR system, the receiver's feedback to the sender is transmitted in the form of a reply message through the same system. In this case, the *receiver's mediator* physical toy presents the emitted message from the receiver in the sender's location operating the tele-puppetry model of communication in the opposite direction. The previous receiver now becomes the sender, and the previous sender, now becomes the receiver.

The *mediator*, who emits the sender's message in a remote location, acts as a focus of the user-interaction for the receiver. Drawing from a framework for information visualization, Kennedy et al. (1996), which has low-latency as a key consideration being derived from a Model–View–Controller (MVC) architectural pattern (Kransner and Pope, 1988), we present the tele-puppetry model of communication as a Model–View–Presenter (MVP) architecture most similar to the definition of Kennedy et al. (1996). In this architectural pattern, the *model* is an interface defining the data to be displayed. The *view* is a passive interface that displays the data. The *presenter* acts upon the model and the view. It retrieves data from the model, and formats it for display in the view.

### 3.2. Media Richness in Intermediated Reality

Fundamental in communication system design is Media richness theory, introduced by Daft and Lengel (1986), which we use to analyse the medium effectiveness of Intermediated Reality. Given the fact that audio, visual and facial cues can be reproduced on a remote intermediary, natural and body languages are seamlessly presented to the remote receiver.

**Figure 3:**
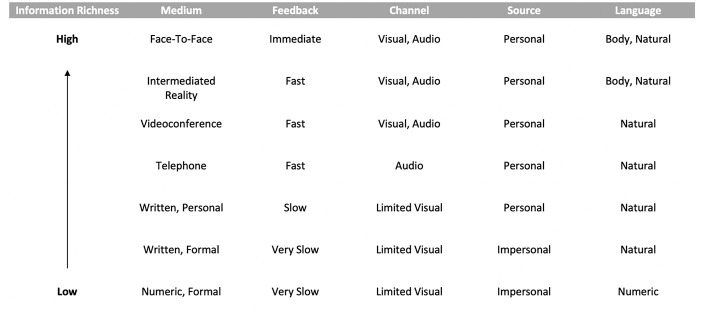
Media richness theory that compares the level of media richness in IR with other types of media.

With the aim of bringing IR experiences closer to a face-to-face experience, we use spatial audio to achieve high fidelity sound when transmitting audible signals. This approach, unlike traditional methods, attaches sound to a specific three-dimensional point in the space (Sodnik et al., 2006). As IR experiences rely on a physical puppet being part of the interaction, we embed the audio source in the upper mouth region of the puppet reproducing the message. As this audio is fully synchronized with the visual and natural cues reproduced on the real-world puppet, our low-latency framework is capable of achieving high medium richness and puts our system one step closer to the pursued instant telepresence (see Figure 3).

## 4. ToyMeet

*ToyMeet* is a CMR system that allows turn based interactions between multiple remote participants using toys brought to life in AR. In order to achieve this, we use *Appearance Retargeting* to bring to life the inanimate body and shadow of static real-world objects (section 4.1). For non-existent geometry in the physical reference model, such as teeth in a closed-mouth real-world model, we use *Appearance Reconstruction* (section 4.2).

**Figure 4:**
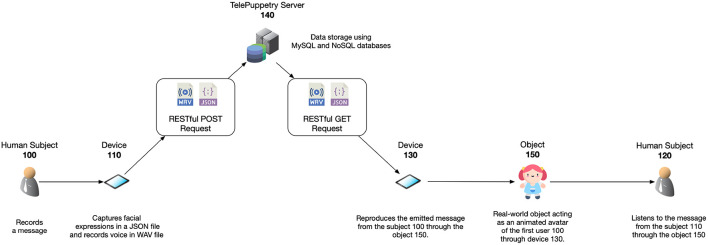
*ToyMeet* system diagram. The sender records a message using his front-facing camera. A message contains voice and facial expressions which are transmitted to a database server. This information is read by the receiver's device afterwards. Upon receiving data, the receiver registers the physical toy acting as a mediator via the rear-facing camera. Once registered, this toy transmits the message using AR.

The sender first sees their own captured sentence locally, live, overlaid, on their selfie viewpoint in AR. The voice and facial expressions get broadcasted to the system's database server (see section 4.3). Next, the receiver's device acquires the transmitted information from this same server and reproduces the content locally using AR in the receiver's physical toy (see section 4.4). This real-world object acts as a the intermediary who reproduces the message recorded by the sender (see Figure 4). Seamless appearance of the physical deformed object is preserved using texture deformation.

### 4.1. Appearance Retargeting

**Figure 5:**
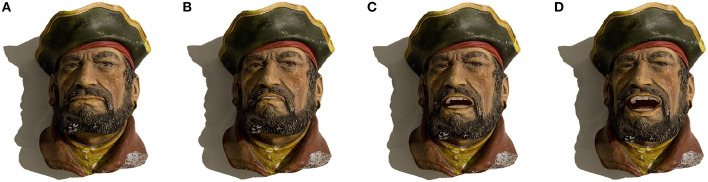
**(A)** Inanimate physical reference toy. **(B–D)** Sequence of frames from a puppet brought to life in AR using *Object Retargeting* and *Shadow Retargeting* (section 4.1) and *Appearance Reconstruction* (section 4.2).

Following the work of Casas et al. (2017), we perform *Object Retargeting* using texture deformation from the real-world toy. This method achieves an illusion of movement from the real-world object through *image retargeting* techniques using Augmented Reality. Such approach allows a photo-realism in AR, since the material, lighting and shading of the virtual object are updated to the mesh in each frame. Any change applied to the real-world environment has its expected lighting effects in the virtual mesh. For the purposes of the augmentation, real-world objects may be static objects or may include moving and movable parts. The animated image of the real-world object may, in some embodiments, be projected onto a portion of the real-world object, such as a head or face portion of an animated puppet or figurine. Figure 5 shows *Object Retargeting* integrated in our CMR system.

In the case for indirect re-lighting, following the work of Casas et al. (2018), we use *Shadow Retargeting*. This method synthesizes virtual shadows directly by sampling the image that contains the shadow of the toy presented in the real-world. As the virtual mesh is animated, this approach warps the real shadow to retarget its appearance to the virtual one. Where the warping samples an area outside the shadow, the appearance gets reconstructed using a search for sampling candidates. This method is of special relevance when facial expressions are integrated into the physical toy changing its appearance and shadow pose. Figure 5 shows *Shadow Retargeting* integrated in our CMR system.

To achieve an accurate registration of the real-world toy, we use marker-less tracking for Augmented Reality using Vuforia (2017). This method consists of a point-based cloud system that recognizes real-world objects. When detected, this point cloud records the origin of the world space coordinates in our framework.

### 4.2. Appearance Reconstruction

For areas not present in the physical reference model, such as teeth and the interior part of the mouth, we need to reconstruct the appearance of the puppet in a way that holds plausible. As our method uses a three-dimensional mesh that matches the shape of the physical object, we can accurately establish the regions that will be revealed and are not visually seen in the reference puppet. Those areas need to be paired with an alike albedo estimate, performed for example beforehand in order to be reconstructed in real-time.

**Figure 6:**
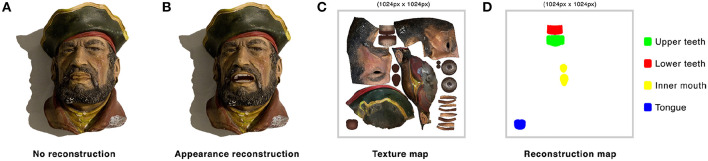
**(A)** Frame of an animated physical puppet in AR without reconstructing occluded areas that become revealed. Teeth and interior parts of the mouth get rendered with the aspect of lips. **(B)** With appearance reconstruction, these parts get rendered plausibly from alike regions of reference. **(C)** Unwrapped texture map of the virtual mesh. **(D)** Color encoded texture map segmenting regions to be reconstructed in real-time.

We unwrap the texture map (Figure 6C) of the model onto the mesh model (Figure 6A). Through the visualization of the mesh in a standard 3d editor, we identify and segment occluded regions of the mesh that have no correspondence on the real-world reference and will need to be reconstructed when blend shapes values are applied to the augmented toy (Figure 6B). We do so by pre-identifying vertices from the geometry that will need to be reconstructed, and hence, *inpainted*. We segment by area according to the element that they represent (Figure 6D). Each element is color encoded in a texture map and paired to an area present in the real-world object that contains a desired similar appearance. This method applies only for cases in which the desired albedo can be sampled from a region that contains a similar appearance. Nonetheless, this in essence generalizes to most puppets and humanoids as these tend to mimic and resemble the outlook and characteristics of humans. Figure 5 shows Appearance Reconstruction integrated in the IR system.

### 4.3. Capturing Sender's Message

In order to allow turn based interactions between multiple remote participants, we need to capture the message being emitted by the sender. To do so, section 4.3.1 describes the procedure employed to capture the user's voice and section 4.3.2 details the acquisition of facial expressions.

#### 4.3.1. Recording Sender's Voice

The sender's voice is recorded with the microphone of their own mobile device. We initialize each sentence recording when the user taps the screen. Once this is tapped for a second time, we finalize the recording. The captured audio is buffered locally on the sender's mobile device and broadcasted to the server of the TMR system once the recording is finished (see section 5.2). Once the file has been broadcasted, the audio buffer is erased from the user's device. We encode the recorded voice using a stereo, 16-bit non compressed Waveform audio file format (*wav*) at 44.100 Hz.

#### 4.3.2. Acquiring Sender's Facial Expressions

To acquire the sender's facial expressions, we use a depth-enabled mobile phone that extracts facial features in real-time using the ARKit (2018). We initialize a recording session when the user taps the button displayed on the screen simultaneously with the voice. For every frame in which the recording session is active, we store the normalized weights of their voice phonemes and facial features for the complete list of attributes). This information is stored locally on the sender's mobile device and broadcast to the server of the IR system once the recording is finished (see section 5.2). Once the data has been broadcast, this animation data buffer is erased from the user's device.

### 4.4. Playing Messages on Animated Puppets

In order to use physical toys as a channel for tele-puppetry, we need to reproduce the message captured by the sender (see section 4.3). Section 4.4.1 describes how the animated meshes of the physical reference object are created. Section 4.4.2 details how these expressions are correctly synchronized with the audio on playback time.

#### 4.4.1. Puppet Facial Expressions

Our method for bringing puppets to life through AR requires a mesh prior for texture deformation. In order to recreate the facial expressions of the sender, we need to reproduce the captured blend-shapes into the puppet's mesh. These consist of 52 key voice phoneme and facial features. To do so, we use a standard 3D editor, such as Autodesk Maya, to create an animated mesh that includes all possible deformations. Each blend-shape is normalized and weighted automatically accordingly with the registered data from the sender.

#### 4.4.2. Adaptive Lip Syncing

As previously introduced in section 4.3.1, we use an uncompressed audio file system to record the user's voice. Specifically, we use the Waveform audio file format, more commonly known as *wav*, due to its use of the Linear Pulse Code Modulation (LPCM) encoding.

Pulse Code Modulation (PCM) is a method used to digitally represent sampled analog signals. In a PCM transmission, the amplitude of the analog signal is sampled regularly at uniform intervals, and each sample is quantized to the nearest value within a range of digital steps. The levels of quantification vary according to the wave amplitude in PCM encodings. However, in a Linear Pulse Code Modulation (LPCM), the quantization levels are linearly uniform within an audio transmission. This linearity allows us to adaptively synchronize the correct facial expression at a given time according to the current LPCM sample. This synchronization is possible because we know the total number of frames recorded for facial expressions and these coincide exactly with the duration of the audio. Such adaptive synchronization is of great importance when the frame rate of the reproduction device differs from the capturing hardware or when the rendering rate fluctuates and does not become constant. This approach does not produce any delay on audio and visual cues as these are always matched to the number of LPCM samples at a current given time. To acquire the current frame (⌊*f*⌋) adaptively, we calculate equation 1 for each rendered frame. (*s*[*t*]) is the number of LPCM samples at a time *t* of the audio clip. (*s*) is the total duration of the audio clip in LPCM samples. (*n*) is the total number of recorded frames that contain facial expressions.

(1) $$\begin{array}{r} \left\lfloor f\right\rfloor =\frac{s\left[t\right]}{\frac{s}{n}} \end{array}$$

## 5. Implementation

In order to use *ToyMeet* as a fluid and smooth collaborative IR system, optimized real-time data processing needs to be leveraged. Section 5.1 details how the sender's facial expressions are serialized over time for an optimal access time. Section 5.2 describes how acquired data is broadcast to a remote server using binary blobs of encoded data for a minimized processing overhead.

### 5.1. Facial Blend Shapes Serialization

As detailed in section 4.3.2, we acquire the sender's facial expressions when we record the emitted message. In order to store the facial blend-shapes sequentially, we serialize their values using the *JavaScript Object Notation (JSON)* file format. When the session is initialized, we allocate a dynamic-sized array to memory. This array gets pushed with a new element on a per-frame basis until the recording finishes. Each element of this array is a dictionary that contains the normalized values of each captured blend-shape.

### 5.2. Performance Broadcast

To optimize for a low-latency communication, we broadcast the serialized facial blend-shapes and the recorded audio messages in a single server request. Our framework streams the content using binary blobs of data in the form of a byte array. This data stream consists of concatenated bytes from the JSON and WAV file using an XML structure. The binary stream of data is transmitted to the server through a web socket that reads chunks of 8,192 bytes at a time. Reading from the streaming continues until the file pointer has either reached the end of file or read the entire byte length. The read data is temporarily stored in a memory buffer on the server. Once the data has been broadcast and buffered on the server, we proceed with the use of relational and non-relational databases to store the data efficiently. In order to so, we first use the XML structure to decode and split the byte array that contains the JSON and WAV files. These get written to the server's disk and labeled using the current timestamp. We then implement a document-oriented database following a NoSQL approach.

With the aim to account for scenarios in which more than two participants interact or communicate with each other, our framework supports broadcasting of audio and visual cues to multiple participants. In this case, one participant of the session takes the role of the host, and all the other ones, subscribe to the session created by that participant. Interactions between peers are labeled, ordered and queued using timestamps, allowing a sequential and natural interaction between participants. Our framework accounts for synchronous and asynchronous sessions as long as two or more participants are not sharing the same physical space, in which case, then, only synchronous interaction can happen. This enables tele-puppetry to a broad range of applications, including, for example, group chats and collaborative interactions.

## 6. Applications

Our framework is designed to account for a diversity of applications in which a collaborative environment can be highly beneficial for the participants. Section 6.1 proposes *ToyMeet* as a tool to enhance storytelling for children using puppets brought to life through mobile Augmented Reality. Section 6.2 presents our framework as a method of telepresence among peers using tele-puppetry as a channel of communication. Section 6.3 presents our technique as a tool in which the puppet's facial expressions can be directed directly from a mobile phone using Augmented Reality.

### 6.1. Compelling Storytelling for Augmented Reality

**Figure 7:**
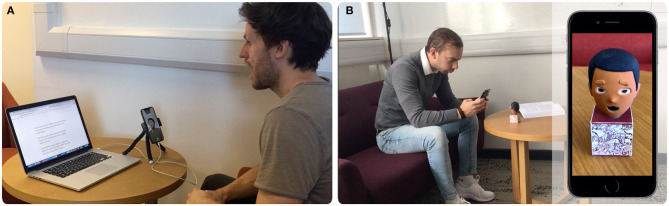
**(A)** *Participant A* recording a story containing voice audio with synchronous facial expressions. **(B)** *Participant B* in a remote location interacting with the recorded story through a toy brought to life in augmented reality.

As Goldstein (1994) evaluated, learning by playing is highly beneficial for the child development. Most toys provide opportunities for children to explore and learn. The most successful are able to capture the children's senses, awaken their imagination and encourage them to interact with others. By using our technique, we can enhance traditional toys with interactive storytelling. We propose to embody the participant into a real-world toy as the narrator of a compelling story (see Figure 7 and Supplementary Material).

### 6.2. Remote Telepresence Among Peers

Positive relationships between parents and children are important for all areas of children's development. By being in the moment, spending quality time and showing warmth, care and respect, the relationship with the child can be strengthening. However, due to the commitments of adults, sometimes parents must be absent for a certain period of time. When this happens, telepresence aims to reduce physical distance by giving the feeling of being in that other location through remote movements, actions or voice. Our technique can make use of augmented reality through traditional toys to reproduce recorded messages of close relatives. This makes both parents and children virtually closer, when in reality, both are far away from each other in the real world. Each of them interacts with other participants through animated physical puppets, helping to awaken the imagination of the child and improving the ability to socially interact with others (see Figure 1 and Supplementary Material).

### 6.3. Fast Facial Posing of Physical Puppets in Stop Motion Animation

Accurately posing stop motion puppets for long-takes movies, frame by frame, is an analog job that requires a high cost in resources and time. Recent approaches aim to reduce these by generating and optimizing in-between frames digitally (Abdrashitov et al., 2018; Casas et al., 2018). Using the same reference model from Casas et al. (2018), we introduce a technique in which we can direct a character's facial expressions directly from a mobile phone using Augmented Reality.

**Figure 8:**
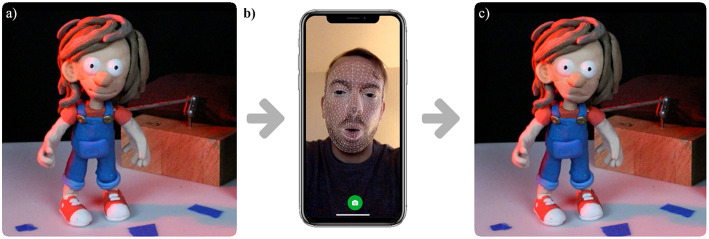
**(a)** Real-world stop motion puppet in an idle pose. **(b)** User posing the facial expressions of a physical puppet using his own mobile phone. **(c)** Directed open mouth in a real-world puppet using photo-realistic AR.

In this work, we propose a method in which a 3D-printed puppet can be directed with the acquired blend-shapes from a user. We use ARKit (2018) to acquire the weighted values from the user and apply them to a rigged and skinned mesh that has the same exact shape as the printed one. This requires to create the 52 key voice phoneme and facial features for each character of a movie. This technique allows high fidelity with the desired result and an accurate synchrony of lips with the recorded audio (see Figure 8 and Supplementary Material).

## 7. Experimental Assessment

Our TMR system targets mobile devices as the platform in which the user experiences mediated communication through tele-puppetry. For such requirement to happen, we stress the importance of an interactive frame rate and low-latency communication. Section 7.1 analyses the result of a comparative user study questionnaire between AR and IR. Section 7.2 provides the result of a System Usability Scale (SUS) evaluation for our TMR system. Section 7.3 analyses the rendering performance in an Apple iPhone X. Section 7.4 describes the system latency for miscellaneous mobile broadbands.

### 7.1. Comparative User Study

In order to measure the effectiveness of communication in an Intermediated Reality system compared to a traditional Augmented Reality one, we conducted a comparative user study between these two technologies. Our study stated the following hypothesis:

“*The interpersonal distance between distributed participants is reduced when using an Intermediate Reality (IR) system compared to a traditional Augmented Reality (AR) one.”*

**Figure 9:**
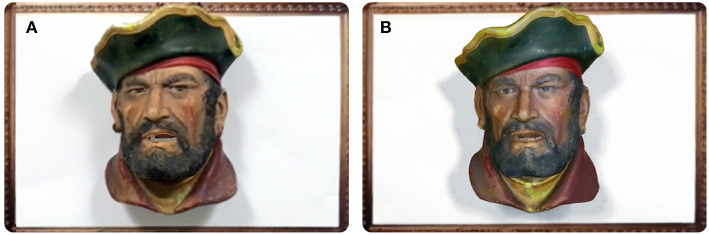
The intermediary that participants got to interact with during the comparative user study in an **(A)** Intermediated reality and **(B)** augmented reality setting.

To evaluate this hypothesis, participants were asked to hold a conversation with a remote participant for 2 min in two different conditions, one being Intermediate Reality and the other being Augmented Reality. For IR, participants were able to interact with the real world object located within a wall-frame through their mobile devices. This object, a plaster's model pirate head (see Figure 9A), came to life and reproduced the remote participant's message. For AR, no real-world object was present on the scene and it was only digitally augmented when participants focused the wall-frame (see Figure 9B). This virtual object reproduced the remote participant's message. Ten participants, 8 male and 2 female, between the age of 22 and 39, undertook the experiment. Within the ten participants, half of them performed the AR condition first and the other half the IR-one first. In both conditions, the conversation took place between a pirate head toy and monkey head toy. The user, who embodied the monkey role, was asked to initiate the conversation by enquiring for a lost treasure. From this point and on, the user had freedom to reply a message with the content and message they pleased. This freedom led participants to be creative in their responses, which was deemed to be an engaging and entertaining experience.

**Figure 10:**
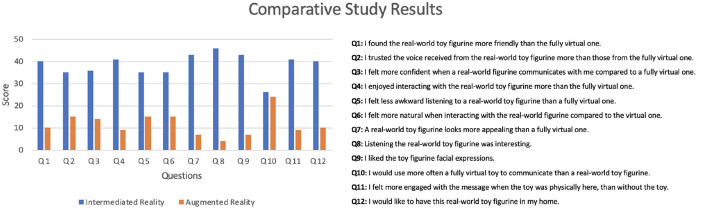
Results of the comparative user study between augmented reality and intermediated reality with ten participants.

Figure 10 shows a transcription of the results of the questionnaire carried out by the participants. Questions were drawn from a related pilot study with life-like robots by Abdollahi et al. (2017). In order to determine if there is a significant difference between IR and AR, we have used a *T-Test* introduced by Gosset (1908). A *t*-test is a type of inferential statistic used to determine if there is a significant difference between the means of two data sets. The calculated result is 0.00000287183, which deems the evaluation to be a positively consistent and statistically significant across all participants.

As seen in the results, users corroborate that an IR-based system is perceived as a richer medium of communication than traditional AR. These stated that a real-world toy figurine using IR is perceived as more friendly and enjoyable that traditional AR. With special outstanding positive feedback, participants found an enlivened real-world toy very interesting and engaging to listen to. They ratified this statement by disapproving the use of a fully virtual toy over an IR-based one. Participants stated that they would use the system equally often with either a virtual or physical intermediary being present. However, in terms of engagement, the system exclusively achieved high evaluation when a physical intermediary was present. This was further emphasized by the fact that participants enjoyed seeing the sender's facial expressions on the their real-world toy figurine. Hence, from this evaluation, users corroborate that the interpersonal distance between distributed participants is reduced compared to traditional Augmented Reality. This improvement is based on the fact that a real-world object, which can be touched and perceived, can embody visual and audible cues with body and natural language from the sender. This phenomenon endorses Intermediated Reality as a medium with high media richness.

At the end of the experiment, participants were openly asked about their perception of the system. They reported to be very engaged with the concept of *Intermediated Reality*, and, overall, found the system novel and exciting for the potential applications it could have. The majority of them stated that they would be interested to use such system if it ever turns to be a commercial viable product.

### 7.2. System Usability Scale (SUS)

Due to the fact that Intermediated Reality proposes a new model of communication not previously presented elsewhere, we understood the urge of analysing the usability of our system from a Human-Computer Interaction point of view. The purpose of this study is to evaluate the end-to-end user experience of our participants as they interact with Intermediated Reality (IR). Collecting this data will provide the study team with behavioral observations and insights into the current user experience, insights into design solutions on how to improve and strengthen the experience and a baseline information on the current experience that can be used as a comparison for future online communication experiences.

We make use of the System Usability Scale (SUS), introduced by Brooke (1996), to evaluate our system with the feedback received from the ten participants, 8 male and 2 female, between the age of 22 and 39, who previously undertook the user study presented in section 7.1. The System Usability Scale (SUS) provides a quick, reliable tool for measuring the usability of a wide variety of products and services. It consists of a 10 item questionnaire with five response options for respondents; from Strongly agree to Strongly disagree. Our system obtains an averaged score across all ten subjects of 79.5. This is the equivalent of a *Good* level of usability according to the SUS adjective scale of Bangor et al. (2009). A SUS score above a 68 is considered to be above average.

**Figure 11:**
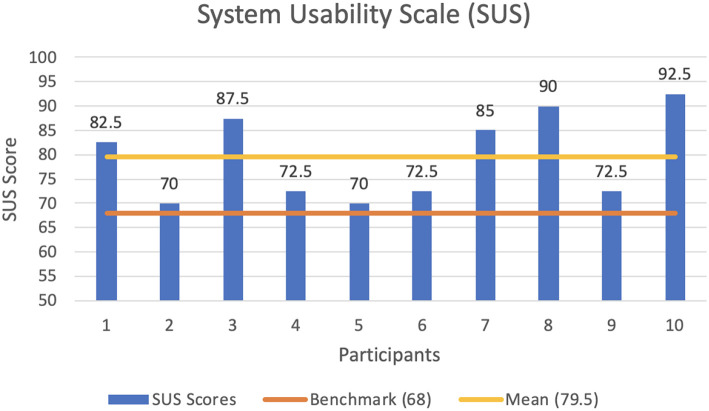
Results of the System Usability Scale (SUS) questionnaire with an average score across all ten subjects of 79.5.

Figure 11 shows a transcription of the results of the questionnaire carried out by the participants. From the results obtained, users state that they would like to use an Intermediated Reality system frequently. They evaluated the framework as easy to use, without many things needed to be learned before being able to use it. However, due to the novel nature of the system, users did not feel totally confident with the system. Such feedback makes us understand that an in-app step-by-step tutorial is needed in order to make the user familiar with an IR system. Nonetheless, they affirmed that most people would be able to learn how to use the system once the IR interaction foundations are in place. Feedback provided by users state that the various functions of the system are well integrated and that IR is an engaging experience. Average scores denotes a very positive outcome, with some margin of improvement on the system usability to achieve an unforgettable experience with final end-users.

### 7.3. Rendering Performance

Our experimental assessment was performed using an Apple iPhone X with an output resolution of 2436 px by 1125 px. We achieved interactive frame rates (23 fps) on this mobile phone.

As seen in Table 1, the primary bottleneck of our system is the *Shadow Retargeting* algorithm. As detailed in Casas et al. (2018), the sampling search for shadow reconstruction requires around 15 ms to achieve coherent results. In order to further optimize this sampling search, the reference-point selection for the discretised concentric ring search algorithm could be further improved by sampling scene visibility. This would better estimate reconstructed shadow appearance. Additionally, this would allow for more advanced techniques to interpolate and even extrapolate the shadow to obtain more consistent results in even more complex scenarios. An approach based on bidirectional reprojection could resolve large deformations or topology changes in which occluder geometry is significantly altered from its physical position (Yang et al., 2011). The impact of such schemes on mobile real-time performance remains uncertain. The rest of time invested in the *Shadow Retargeting* algorithm is for shadow warping, auxiliary masks and Percentage Closer Soft Shadows (PCSS). On average, retargeting the shape of the shadow takes approximately two thirds of the render time per frame.

**Table 1:** Time breakdown of a typical frame processed using *ToyMeet* in an Apple iPhone X.

**Task**	**Time (ms)**	**Percentage**
AR marker-less tracking	1.85	3.91
Object retargeting	4.87	10.31
Shadow retargeting	34.13	72.26
Appearance reconstruction	2.64	5.59
Background inpainting	2.78	5.88
Scene rendering	0.96	2.03
Total	46.9	100

The remainder third of the render time per frame is invested in miscellaneous duties. The second substantial task, which takes around 10% of the rendering time per frame, is object retargeting. This section encapsulates duties such as transferring texture data from the camera feed, rendering the overlaid mesh in a deformed position or assigning the weighted blend-shapes in real-time. Following this task, with an approximate 5% of the rendering time per duty, appearance reconstruction for occluded areas and background inpainting over uniform backdrops rank. Finally, marker-less AR tracking and scene rendering take the remainder 5% of the rendering time per frame.

### 7.4. System Latency

Our framework is optimized for low-latency communications among participants. As *ToyMeet* is designed to work efficiently in mobile devices, we analyse the broadcasting times in different broadbands. In Table 2, we breakdown the data size per frame, which can then be extrapolated for each second or by a sample message of 10 s. Each serialized JSON blend-shape frame takes the size of 2,045 bytes. This includes the normalized weighted values of the 52 key phoneme and facial features and the position and rotation of the captured face in world coordinates. In addition to the blend-shapes, we record the syncronized audio using a stereo, 16-bit non-compressed WAV recording at 44,100 KHz. This has a bit-rate of 1,411.2 Kbps, which sizes at 5,880 bytes per frame. The combined captured data amount is 7,925 bytes per frame.

**Table 2:** File size breakdown analyzed per frame, second and 10 s.

**File type**	**Bytes/frame**	**KB/second**	**MB/10 s**
JSON data	2,045 bytes	61.35 KB	0.613 MB
WAV audio	5,880 bytes	176.4 KB	1.764 MB
Total	7,925 bytes	237.75 KB	2.377 MB

*Captured frame-rate is calculated at 30 fps. Recorded audio files are stereo, 16-bit at 44,100 KHz*.

Seven thousand nine hundred and twenty five bytes per frame may seem like a small number, but when done at 30fps in a slow broadband, the transmission time can be a challenge. As it can be seen in Table 3, this is specially the case for GPRS (2.5G) and EDGE (2.75G) connections, in which a sample message of 10 s could take almost 3 min to be broadcasted. This is not the case for faster connections, such as HSPA (3G) or LTE (4G). In this case, data transmissions are well optimized and broadcasting times are as little as 0.38 s for a sample message of 10 s in a LTE broadband. With the upcoming plans for 5G connections, with guaranteed minimum speeds of 10 GBps, our framework will accomplish remote synchronous real-time capabilities. Hence, we understand that for a smooth and low-latency communication the user should have at least a HSPA (3G) broadband. Currently, such connection has a penetration of 85% worldwide.

**Table 3:** Time breakdown for broadcasting combined recorded audio and serialized blend-shapes in miscellaneous mobile broadbands analyzed per frame, second and 10 s.

**Broadband**	**Speed**	**Frame**	**1 s**	**10 s**
GPRS (2.5G)	0.115 Mbit/s	0.55116 s	16.535 s	165.35 s
EDGE (2.75G)	0.237 Mbit/s	0.26743 s	8.023 s	80.23 s
HSPA (3G)	5.8 Mbit/s	0.01090 s	0.327 s	3.27 s
LTE (4G)	50 Mbit/s	0.00126 s	0.038 s	0.38 s
WiFi	100 Mbit/s	0.00063 s	0.019 s	0.19 s
eMBB (5G)	10 Gbit/s	0.000063 s	0.0019 s	0.019 s

*Calculated times do not take into account accidental lost packages caused by the user's environment, such as packet collision, radio interference or over-demanded service*.

## 8. Limitations And Future Work

Our current system is capable of providing communication asynchronously. When the sender records a message, it is sent first through the Tele-Puppetry server, then it is played by the remote participant and then a response is created. It is not necessary for this to happen at the exact same time, and minutes or hours may elapse between messaging loops. This is the same approach to communication that mobile messages or Walkie-Talkies have. In our future work, our goal is to be able to provide synchronous and asynchronous communication and let the user decide which one best suits their needs for a specific occasion or task. An important technical challenge that must be overcome is the ability to track the face through the front camera of the mobile phone and play the contents of an AR toy from the rear camera at the same time in iOS. Currently, there are only some Android phones on the market that support dual camera use at the same time, such as the Nokia 6.1 Android phone with *Bothie Mode* or the Samsung Galaxy S8. With the raise of AR support in iOS devices, this should be overcome in the near future. A concurrent system, however, would further require a solution to address live 2-way streams of voice and animation data. Low-latency speeds can enhance the security of the framework by enabling distributed encryption protocols, such as implementations based on block-chain algorithms. Our focus on compact efficient data transmission lends itself to this scenario in future work.

Our system requires having a three-dimensional version of the real-world object with all blend-shapes modeled in order to create AR toy figures. This task is not simple and needs specific adjustments for each model, which requires an artist to manually modify each expression for each model. We anticipate that we will be able to reduce the time dedicated to this task by creating a set of predefined expressions that can be retargeted to new models by transferring the weights of the facial bones in the 3D model. This would set the foundations for a potential auto-rigging and skinning system that would speed up the animation process of organic and non-organic objects. Such approach would reduce the time needed to create blend-shapes, since the only adjustment required would be to fine-tune parameters according to each 3D model.

We foresee future user studies being conducted around social tele-presence. We anticipate collecting observational and conversational data in order to further analyse and discuss the results obtained in the comparative user study detailed in section 7.1. We plan on conducting informal interview questions to gather extensive feedback from participants.

In applying our concept more broadly, we foresee applications of our framework across the entire reality-virtuality continuum. Using full immersive Virtual Reality, in which the physical intermediary is not seen, we anticipate the use of haptics systems and virtual characters for getting in touch with the receiver's physical space, Bierz et al. (2005). In a robotic telepresence scenario, in which the intermediary is a robot with spatial audio systems and physical animated facial expressions, we envision the robot to be driven by our system using synchronized audio and captured facial data. In addition, we see the intermediary element of our communication model with the capability to be a Time Traveler Proxy (TTP). As proposed by Tang et al. (2012), this would allow participants who are unable to attend a meeting to use pre-recorded messages to interact with other members. Using our system, *ToyMeet*, this could be done through a real-world delegate with synchronized audio and captured facial expressions. Hence, we understand that *Intermediated Reality* has a broad scope of applications in miscellaneous industrial sectors.

## 9. Conclusion

In this research paper, we introduced *Intermediated Reality*. Our collaborative tele-present Mixed Reality system is the first to propose a framework for mediated communication through the remote possession of toys that come to life in mobile Augmented Reality. Our approach has shown how, as part of a two-way conversation, each person communicates through a toy figurine that is remotely located in front of the other participant. Each person's face is tracked through the front camera of their mobile devices and the tracking pose information is then transmitted to the remote participant's device along with the synchronized voice audio, allowing a turn-based interaction chat.

Additionally, we have demonstrated how such a system could be used as a tool for enhancing storytelling to children using puppets brought to life in AR. We have proposed to embody a narrator into the enlivened toy to enrich creativity in children. Finally, we have showcased how our framework could rapid pose facial expressions in real-world puppets using AR. Our method would allow reductions in costs and time allowing a fast facial posing method for Stop Motion movies.

## Data Availability

The datasets generated for this study are available on request to the corresponding author.

## Ethics Statement

Ethical approval was obtained from the local ethics committee composed by Kevin Chalmers, Gregory Leplatre, and Ben Paechter affiliated at Edinburgh Napier University. Written informed consent was obtained from participants for involvement in the experiment and publication of their image.

## Author Contributions

LC and KM contributed to the design and implementation of the research, to the analysis of the results and to the writing of the manuscript. KM contributed to the creation of artistic assets.

### Conflict of Interest Statement

The authors declare that the research was conducted in the absence of any commercial or financial relationships that could be construed as a potential conflict of interest.
